# Sintilimab-Induced Autoimmune Diabetes in a Patient With the Anti-tumor Effect of Partial Regression

**DOI:** 10.3389/fimmu.2020.02076

**Published:** 2020-08-21

**Authors:** Liang Wen, Xiuwen Zou, Yiwen Chen, Xueli Bai, Tingbo Liang

**Affiliations:** ^1^Department of Hepatobiliary and Pancreatic Surgery, The First Affiliated Hospital, Zhejiang University School of Medicine, Hangzhou, China; ^2^Zhejiang Clinical Research Center of Hepatobiliary and Pancreatic Diseases, Hangzhou, China; ^3^Zhejiang Provincial Key Laboratory of Pancreatic Disease, The First Affiliated Hospital, Zhejiang University School of Medicine, Hangzhou, China; ^4^The Innovation Center for the Study of Pancreatic Diseases of Zhejiang Province, Hangzhou, China

**Keywords:** PD-1 inhibitor, Sintilimab, hepatocellular carcinoma, immune related adverse event, autoimmune diabetes, plasma glucose

## Abstract

**Context:**

Immune checkpoint blockades (ICBs) have been approved widely to treat various malignancies. Autoimmune diabetes mellitus, which can be caused by programmed cell death protein 1 (PD-1) inhibitors, is rare. Sintilimab, a monoclonal anti-PD-1 antibody, has been approved in China for the treatment of Hodgkin’s lymphoma and was used in our clinical trial for patients with unresectable hepatocellular carcinoma (HCC).

**Case Presentation:**

We present the first case of autoimmune diabetes during Sintilimab treatment in a patient with unresectable HCC, accompanied by a remarkable anti-tumor effect of partial regression. A 56-year-old male with typical symptoms presented with diabetic ketoacidosis (DKA) at 24 weeks after Sintilimab initiation. His fasting plasma glucose level was 22.2 mmol/L, HbA1c was 7.8%, fasting insulin was 1.5 mIU/L, and fasting C-peptide was 1.12 ng/mL, which further decreased to 0.21 ng/mL 4 days later. The patient was diagnosed with new-onset diabetes mellitus using the oral glucose tolerance test. The anti-glutamic acid decarboxylase 65 antibody, anti-islet cell antibody, and anti-insulin antibody tests were all negative. For the type 1 diabetes-associated alleles of human leukocyte antigen (HLA) class I and II, the most relevant type was identified as HLA-A^∗^0201. A diagnosis of PD-1 inhibitor-induced autoimmune diabetes was made. After rectification of DKA, he was treated with insulin therapy daily, which has since controlled his plasma glucose well. Thereafter, Sintilimab was been continued with sustained therapeutic effect.

**Conclusion:**

Due to unpredictability of this rare immune related adverse event (irAE), diabetes-related autoantibodies and C-peptide are recommended to be tested before immunotherapy, and plasma glucose monitoring should be performed. After plasma glucose is well controlled using insulin therapy, PD-1 inhibitor treatment might be continued, especially when the immunotherapy is effective.

## Introduction

Immune checkpoint blockades (ICBs) have been approved widely to treat various malignancies. ICBs interfere with the inhibitory signals of T cell activation, and restore the function of adaptive immune response to enhance the anti-tumor effect. However, activated immune cells sometimes damage normal tissues and cause a series of immune-related events, including dermatological, pulmonary, gastrointestinal, cardiovascular, and endocrine adverse effects (1). In addition to other immune-related adverse events (irAEs), hypophysitis and thyroid dysfunction are the common endocrinopathies. However, autoimmune diabetes mellitus, usually caused by programmed cell death protein 1 (PD-1)/programmed cell death ligand-1 (PD-L1) inhibitors, is rare and seldom reported (2), especially in patients with liver cancer.

Sintilimab is a fully human IgG4 monoclonal antibody that binds to PD-1 that has been approved in China to treat patients with relapsed or refractory Hodgkin’s lymphoma (3). To extend its potential therapeutic effect in other tumors, we sponsored a clinical trial, CISLD-1 (NCT03732547), one of the aims of which was to evaluate the therapeutic safety and efficacy of Sintilimab for patients with unresectable hepatocellular carcinoma (HCC). The present study reports a case of new-onset autoimmune diabetes in a patient with HCC during treatment with Sintilimab, accompanied with remarkable anti-tumor therapeutic efficacy.

## Methods

This study was carried out in accordance with the recommendations of the Center for Ethics in First Affiliated Hospital of Zhejiang University. Written informed consent from the subject was obtained for the publication of this case and any potentially identifying images or information.

## Case Presentation

**FIGURE 1:**
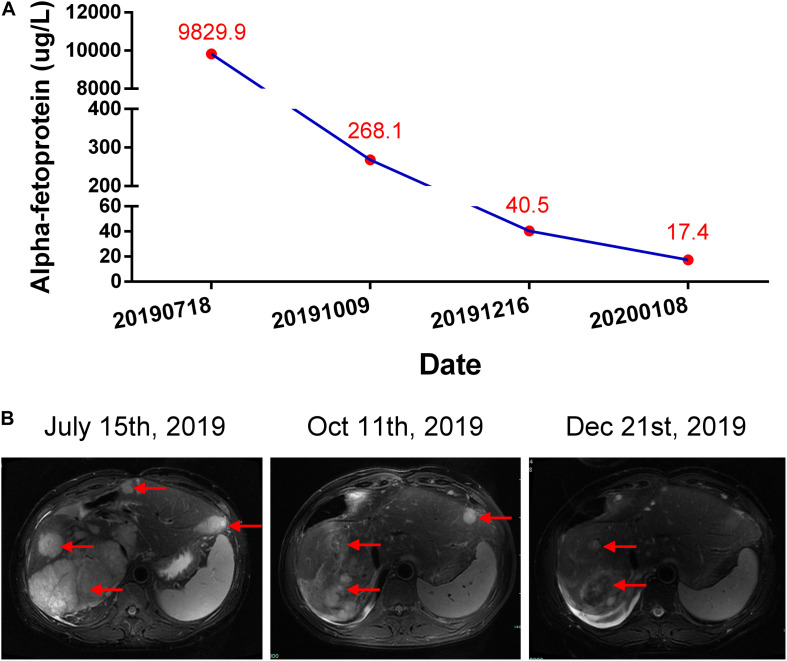
Courses of the patient’s treatments. **(A)** Changes of alpha-fetoprotein (AFP) level during each period. **(B)** Representative hepatic magnetic resonance (T2 weighted) assessments during each period. Red arrows indicate the tumors.

A 56-year-old male was diagnosed with multiple HCCs in Barcelona Clinic Liver Cancer (BCLC) stage B. The patient had chronic viral hepatitis B and received standard antiviral therapy. He had no medical history of glucose intolerance, autoimmune diseases, thyroid dysfunction, or other systemic diseases (e.g., Cushing’s syndrome), and no evidence of acute infection, trauma, or drug poisoning. He also had no family history of diabetes, autoimmune diseases, and hereditary diseases. Psychosocial assessment showed that the patient had no mental, physical, or emotional health issues. After unsuccessful treatment with trans-hepatic arterial chemotherapy and embolization (TACE), the patient voluntarily participated in the CISLD-1 trial, provided signed informed consent in July 2019, and started to receive Sintilimab at a dose of 200 mg every 3 weeks. Five months after receiving Sintilimab, tumor marker examination and imaging scans showed that the treatment was effective. The patient’s alpha-fetoprotein (AFP) level decreased to 40.4 μg/L from a baseline level of 9829.9 μg/L (Figure 1A). Magnetic resonance imaging (MRI) showed that the tumor nodule in the liver had shrunk dramatically with a response evaluation of partial regression (PR) according to Response Evaluation Criteria in Solid Tumors (RECIST) version 1.1 (Figure 1B). During these months, the patient felt mild fever regularly; however, there was no fatigue, nausea, erythema or any other side effect found.

**FIGURE 2:**
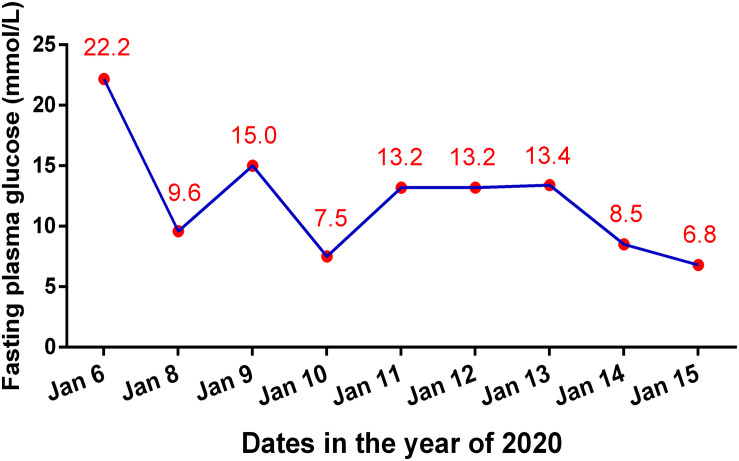
Changes of fasting plasma glucose level during the hospital stay because of diabetic ketoacidosis.

At 24 weeks, after 8 courses of Sintilimab, the patient presented with increased urination and drinking for 1 week, and blood tests showed that his fasting plasma glucose was as high as 22.2 mmol/L, with an HbA1c value of 7.8%. Considering the high risk of diabetic ketoacidosis, blood gas analysis was performed, which revealed that he had severe metabolic acidosis, with an arterial pH of 7.27, serum bicarbonate of 12.9 mmol/L, and lactate of 1.8 mmol/L. The blood ketone body determination was positive. Therefore, the patient was suspected to have new onset diabetes mellitus with ketoacidosis and was admitted to our hospital. Further blood assessment showed that the patient’s fasting insulin was as low as 1.5 mIU/L and fasting C-peptide was 1.12 ng/mL (0.78–5.19 ng/mL), which further decreased to 0.21 ng/mL 4 days later. The oral glucose tolerance test (OGTT) revealed that the fasting, 1-, 2-, and 3-h plasma glucose levels were 12.34, 18.32, 28.38, and 25.41 mmol/L, respectively, accompanied by a 2-h postprandial C-peptide level of 0.30 ng/mL and a serum insulin level of 10.4 mIU/L. In addition, the patient’s serum inflammatory cytokines, measured using an enzyme-linked immunosorbent assay (ELISA), demonstrated an interleukin-6 (IL-6) level of 786.32 pg/mL, which decreased to 338.70 pg/mL 2 months later (Table 1). There was no infection, medication, thromboembolic event, or other factor (Autoimmune diseases, Cushing’s syndrome, or drug poisoning) that could cause hyperglycemia. Consequently, Sintilimab-induced new-onset autoimmune diabetes was diagnosed. However, the anti-glutamic acid decarboxylase 65 (GADA) antibody, anti-islet cell antibody (ICA), and anti-insulin (IAA) antibody tests were all negative. In addition, the type 1 diabetes-related alleles of human leukocyte antigen (HLA) class I and II, which were explored at the loci of HLA-A, B, C, DRB1, DQA1 and DQB1 by sequence-based typing primed PCR (Table 2), revealed that the most relevant allele was HLA-A^∗^0201 (4). For other endocrine function assessments, thyroid hormones showed that the patient’s serum total triiodothyronine (TT3) and free triiodothyronine (FT3) were slightly decreased, but later become normal without oral thyroxine treatment (Table 1). Moreover, the levels of anterior pituitary hormones and their regulated hormones were all normal (Table 1). The patient received insulin therapy delivered by micropump, maintaining water and electrolyte acid-base balance and other supportive treatment to correct acidosis. Thereafter, he was treated with insulin therapy subcutaneously, which was adjusted daily to the dose of once-daily basal insulin glargine (long-acting insulin, 30 units) plus thrice-daily prandial insulin aspart (fast acting insulin, 14 units) during his hospital stay. The patient’s plasma glucose returned to normal levels gradually and he was discharged 10 days later (Figure 2). Subsequently, the patient’s plasma glucose was monitored in the outpatient setting and the insulin therapy was gradually adjusted to once-daily basal insulin glargine (28 units) plus thrice-daily prandial insulin aspart (15 units). Then, the patient received continued Sintilimab administration, which resulted in a further decreased in his AFP level (Figure 1A). And the patient’s plasma glucose level was stable during follow-up visits.

**TABLE 1:** Levels of hormones and inflammatory cytokines before and after initiation of Sintilimab.

**Hormones**	**Before Sintilmab initiation**	**24 weeks after Sintilmab initiation**	**31 weeks after Sintilmab initiation**
TSH (0.350–4.940 mIU/L)	1.272	0.656	1.35
FT4 (9.01–19.05 pmol/L)	16.40	13.60	16.81
FT3 (2.63–5.70 pmol/L)	3.62	2.21	4.61
TT4 (62.68–150.84 nmol/L)	212.03	90.24	125.90
TT3 (0.89–2.44 nmol/L)	1.59	0.76	1.90
PTH (15.0–65.0pg/mL)	No data	13.7	No data
Testosterone (142.39–923.14 ng/dL)	No data	105.38	No data
Estradiol (11.00–44.00 ng/dL)	No data	32.56	No data
FSH (0.95–11.95 mIU/mL)	No data	11.06	No data
LH (0.57–12.07 mIU/mL)	No data	6.46	No data
Prolactin (3.46–19.40 ng/mL)	No data	17.37	No data
Progesterone (0.00–0.20 ng/mL)	No data	<0.10	No data
HGH (0.00–3.00 ng/mL)	No data	0.31	No data
8 am cortisol (μg/dL)	No data	14.50	No data
8 am ACTH (pg/mL)	No data	27.80	No data
IL-1β (0.10–80.0 pg/mL)	No data	64.72	68.64
IL-2 (0.10–4.10 pg/mL)	No data	0.10	0.10
IL-4 (0.10-3.20 pg/mL)	No data	0.10	0.10
IL-6 (0.10–2.90 pg/mL)	No data	786.32	338.70
IL-10 (0.10–5.00 pg/mL)	No data	0.77	2.22
TNF-α (0.10–23.00 pg/mL)	No data	0.30	0.36
IFN-γ (0.10–18.00 pg/mL)	No data	0.10	0.10
IL-17A (0.10–2.90 pg/mL)	No data	0.10	0.10

*TSH, thyroid stimulating hormone; FT4, free thyroxine; FT3, free triiodothyronine; TT4, total thyroxine; TT3, total triiodothyronine; PTH, Parathyroid hormone; FSH, follicle-stimulating hormone; LH, luteinizing hormone; ACTH, adrenocorticotropic hormone; HGH, human growth hormone; IL, interleukin; TNF-α, tumor necrosis factor alpha; IFN-γ, interferon gamma.*

**TABLE 2:** Identified alleles of human leukocyte antigen (HLA) class I and II at the selected loci.

**HLA locus**	**A**	**B**	**C**	**DRB1**	**DQB1**	**DQA1**
Alleles	0201, 2403	1525, 4002	0304, 0403	1201, 1202	0503, 0301	0104, 0601

## Discussion

Sintilimab, a monoclonal antibody, is an ICB that targets to PD-1, thus blocking the pairing of PD-1 and PD-L1/PD-L2. In the tumor microenvironment, tumor cells usually express PD-L1 to activate inhibitory costimulatory signals by coupling with PD-1 in lymphocytes, which leads to T cell tolerance or even exhaustion, resulting in a failed anti-tumor response in the host. However, in addition to tumor cells, PD-L1 is also widely expressed in cells of normal tissues, including the insulin producing beta-cells in the islets of the pancreas (5, 6). Thus, PD-1-PD-L1 blockade sometimes harms protective autoimmunity and produces tissue damage via activated lymphocytes, which can result in irreversible beta-cell destruction in autoimmune diabetes. Animal studies have revealed that PD-1/PD-L1 deficiency or blockade activates T cells infiltrating into islets and results in selective beta-cell destruction in non-obese diabetic (NOD) mouse models (7, 8). Evidence also shows decreased PD-1 expression in circulating T cells in patients with new-onset type 1 diabetes following anti-PD-1 and anti-PD-L1 treatment (9, 10). By contrast, several proinflammatory cytokines, especially tumor necrosis factor alpha (TNF-α), interleukin 1 beta (IL-1β), and interferon gamma (IFN-γ), have been shown to play important roles in developing type 1 diabetes at the level of both the immune response and targeting beta-cells (11). However, in this patient, instead of TNF-α, IL-1β, and IFN-γ, only IL-6 was dramatically elevated, which indicated the particularity of PD-1 inhibitor-induced diabetes. Thus, the precise mechanism mediating ICB-related autoimmune diabetes remains to be further explored.

Autoimmune diabetes is a rare type irAE induced by ICBs. Pembrolizumab (Keytruda) and nivolumab (Opdivo), the earliest approved PD-1 inhibitors, lead to the development of autoimmune diabetes at a rate of 0.2–0.9%, which is much lower than other irAEs, including pneumonitis, colitis, hepatitis, dermatitis, and nephritis (12). In a retrospective review summarizing 42 cases of PD-1 inhibitor (Pembrolizumab)-induced type 1 diabetes mellitus (12), the authors concluded that the median time from initiation of anti-PD-1 therapy to development of diabetes mellitus was 6 weeks (ranging from 1 to 52 weeks). In this case, the patient developed diabetes mellitus at 24 weeks. Unlike the classical type 1 diabetes, which is usually diagnosed in young patients, PD-1 inhibitor-induced diabetes could occur at any age. Most of patients present with diabetic ketoacidosis in a short time, with decreased or undetectable serum C-peptide and a moderately low HbA1c level during follow-up. For diabetes related autoantibodies, their presence at baseline is a risk factor for the onset of PD-1 inhibitor-induced type 1 diabetes in patients. In addition, half of the patients with type 1 diabetes were positive for GADA, which was always accompanied by positivity for other antibodies. In our case, the patients was negative for GADA, ICA, and IAA. For the genetically susceptible HLA I and II genes, many specific alleles at the loci of DRB1, DQA1, and DQB1 and the haplotypes of DR3-DQ2 and DR4-DQ8, are strongly associated with type 1 diabetes (13, 14). However, we did not find these high risk alleles or haplotypes in this patient. Instead, the allele of HLA A^∗^0201 in this patient was reported to be relevant with diabetes susceptibility (4), which has not been reported in the onset diabetes by anti-PD-1 treatment. In addition, studies about genetic polymorphisms within *PDCD1* (encoding PD-1) identified single nucleotide polymorphisms (SNPs) of rs111568821, rs2227981, rs2227982, and rs4143815 as significantly associated with type 1 diabetes susceptibility (15, 16). Combined ICB treatment might also carry an increased risk of diabetes compared with that of monotherapy. Considering the multiple risk factors and unpredictability of autoimmune diabetes by anti-PD-1 treatment, we recommend that the baseline levels of diabetes-related autoantibodies, C-peptide, and HLA class gene patterns are tested, and regular plasma glucose monitoring before and after immune therapy should be included in the routine examination.

As it is for type 1 diabetes, long-term exogenous insulin injection should be the basic treatment for PD-1 inhibitor-induced diabetes after rectification of potential diabetic ketoacidosis. In contrast to other irAEs caused by ICBs, including pneumonia, nephritis, and myocarditis., for autoimmune diabetes mellitus, immunosuppression with corticosteroids is not helpful, since most of the beta-cells in the islets will be irreversibly destroyed (17), and most reports stated that there was no remissions of diabetes after cessation of PD-1 inhibitor treatment. Only one case reported the reversal of insulin-dependence in a patient with residual beta-cell function (18). Further studies are needed to test if other immunosuppressive agents could be used to reduce the damage to beta-cells. Once the plasma glucose is controlled well, it is reported PD-1 inhibitors can be restarted unless disease progression or other drug toxicity occurs (17). In our case, apart from treatment with multi-subcutaneous insulin injection, we did not use any immunosuppressive agents. As for the thyroid hormones indicators, low serum TT3 and FT3 levels self-normalized without oral thyroxine treatment, which was thought to be a low T3 syndrome accompanied by diabetic ketoacidosis (19). Our patient continued to receive Sintilimab soon after good control of his hyperglycemia was achieved. Under regular insulin treatment and good alimentary control, plasma glucose monitoring showed that immunotherapy did not cause more fluctuations in plasma glucose. The patient’s AFP level further decreased, revealing the duration of therapeutic efficacy.

In this case, although the patient had the drug related adverse event of autoimmune diabetes and mild fever, fortunately he also achieved an anti-tumor response of PR according to RECIST version 1.1. There are reported associations between the frequencies of adverse events and the therapeutic efficacy of ICBs. Some reports showed that in advanced cancer treated with PD-1 inhibitors, patients with irAEs showed a markedly better efficacy over patients without irAEs, including a higher overall response rate (ORR), prolonged progression free survival (PFS), and longer overall survival (OS) (20, 21). No study has focused on the relationship between new-onset autoimmune diabetes and the therapeutic efficacy of PD-1 inhibitors. However, we could speculate that because of the excessive activation of adaptive immunity in the patient with autoimmune diabetes, it is reasonable that the anti-tumor response tends to be more efficient. Therefore, based on the controlled drug toxicity in immunotherapy, continued treatment with the PD-1 inhibitor should be considered if an efficient anti-tumor response is observed.

## Conclusion

Immune checkpoint inhibitors, especially PD-1 inhibitors, cause autoimmune diabetes, usually accompanied with severe complications like ketoacidosis. The unpredictability of this rare irAE suggests that the baseline levels of diabetes-related autoantibodies and C-peptide are considered to be tested, and regular plasma glucose monitoring should be added to the routine examination. Based on the good plasma glucose control achieved using basic insulin therapy, PD-1 inhibitor treatment is considered to be continued, especially when the immunotherapy is effective in treating the patient’s malignancy.

## Data Availability Statement

The raw data supporting the conclusions of this article will be made available by the authors, without undue reservation, to any qualified researcher.

## Ethics Statement

The studies involving human participants were reviewed and approved by Center for Ethics in First Affiliated Hospital of Zhejiang University. The patients/participants provided their written informed consent to participate in this study. Written informed consent was obtained from the individual(s) for the publication of any potentially identifiable images or data included in this article.

## Author Contributions

LW analyzed the patient data, designed the case report, and drafted the manuscript. XZ provided significant contributions to the collection of patient data and sample preparation. YC provided significant contributions to the analysis and interpretation of MRI imaging. XB and TL provided significant contributions to the analysis of the patient data and designed the case report. All authors read and approved the final draft of the manuscript.

## Conflict of Interest

The authors declare that the research was conducted in the absence of any commercial or financial relationships that could be construed as a potential conflict of interest.
